# Proton Beam Therapy for Unresectable Mediastinal and Pericardial Spindle Cell Sarcoma: A Case Report

**DOI:** 10.14338/IJPT-23-00001.1

**Published:** 2023-05-09

**Authors:** Brady S. Laughlin, Joshua Stoker, Tamara Vern-Gross

**Affiliations:** Department of Radiation Oncology, Mayo Clinic, Phoenix, AZ, USA

**Keywords:** sarcoma, proton beam therapy, radiation toxicities

## Abstract

Unresectable mediastinal soft tissue sarcomas are often aggressive and associated with a poor prognosis. A 17-year-old male presented with progressive fatigue, shortness of breath, and heart palpitations secondary to an extensive mass involving the mediastinum and pericardium. He was treated with chemotherapy per protocol Children’s Oncology Group Protocol ARST0332 and proton beam therapy to the involved mediastinum, pericardium, and heart. At the 5-year follow-up evaluation, he remained disease-free on surveillance imaging. An echocardiogram revealed a 55% to 60% left ventricular ejection fraction. Given the patient’s extended survival, we present the oncologic rationale for treatment and considerations of late toxicity.

## Introduction

Soft tissue sarcomas are malignant tumors of mesenchymal origin, which may present in many anatomic locations [1]. Primary sarcomas in the mediastinum are extremely rare; they account for 2% of all soft tissue sarcomas and less than 10% of primary mediastinal tumors [2–4]. With the lack of standard guidelines, many mediastinal sarcoma tumors present as locally advanced or unresectable, making their management complex [5]. In general, mediastinal soft tissue sarcomas have a poor prognosis with a mean survival ranging from 4 to 34 months [2, 5–9]. Patients who cannot undergo complete resection or have an unresectable disease have poor prognoses [2]. In this case study, we present the clinical diagnosis and treatment of a 17-year-old male with unresectable high-grade spindle cell sarcoma involving the mediastinum and pericardium. Radiation therapy is standardly used for unresectable or incompletely resected sarcoma. The rationale for proton beam therapy was that a local treatment could be provided while delivering less radiation to adjacent lungs, thereby decreasing the risk of acute and late radiotherapeutic toxicities.

## Case Report

A 17-year-old male initially presented to his primary care provider with a 2-month history of intermittent heart palpitations, shortness of breath, and progressive fatigue, for which he was referred to a cardiologist who obtained an echocardiogram (ECHO). This revealed a pericardial effusion. He was initially started on ibuprofen 1800-mg daily and sent home. He returned 2 weeks later for a repeat ECHO, and the pericardial effusion had increased, leading to furosemide administration. An ECHO at the next follow-up visit 2 weeks later noted the resolution of the effusion, and the diuretic and anti-inflammatory medication were discontinued. He subsequently presented to an emergency department with right-sided chest pain, dyspnea upon exertion, easy fatigability, decreased appetite, and difficulty sleeping. An electrocardiogram and ECHO revealed restrictive pericarditis. Prednisolone was initiated, and he was hospitalized for further management in the Pediatric Intensive Care Unit. Chest X-ray revealed a large cardiac silhouette. Dedicated computed tomography (CT) of the thorax revealed 3 low-attenuation masses involving the anterior and middle compartments of the mediastinum and the pericardium. The largest tumor, measuring 6.2 × 8.7 × 8.8 cm, surrounded the descending aorta, a portion of the transverse aortic arch, the main pulmonary artery, and the right and left main pulmonary arteries. There was a notable narrowing of all 4 pulmonary veins. A focal retrocardiac mass was visualized surrounding the right lower pulmonary vein, measuring 4.3 × 2.9 × 3.8 cm. Additionally, a smaller retrocardiac mass surrounding the left lower pulmonary vein measured 2.5 × 2.4 × 2.1 cm with associated thickening of the pericardium. Magnetic resonance imaging (MRI) of the heart demonstrated nodular thickening of the pericardium with narrowing of the right and left main pulmonary arteries and pulmonary veins with a mass surrounding the ascending aorta and anterior mediastinum.

After this initial imaging, the patient was treated for restrictive pericarditis, peripheral edema, and congestive heart failure. Further staging studies included CT imaging of the pelvis and whole-body position-emission tomography/CT, which revealed no evidence of lymphadenopathy, suspicious osseous lesions, or metastases. After a work-up for possible lymphoma or germ cell tumor, the patient underwent a Chamberlain procedure and open biopsy of the mediastinal mass. The pathology report indicated the tumor was a high-grade spindle cell sarcoma, NOS (AJCC clinical T2b, NX, M0). The specific line of differentiation could not be identified. The immunophenotype was inconsistent with rhabdomyosarcoma (desmin negative), and fluorescence in situ hybridization studies did not support synovial sarcoma (negative for translocation involving SS18). Mitotic activity was greater than 20 per 10 high-power fields, and the focus of necrosis involved less than 50% of the tumor. Immunohistochemical studies demonstrated that the cells were positive for vimentin and CD117, weakly positive for BCL-2 and CD34, and negative for desmin, CD99, S100, DOG1, CD45, EMA, Cam5.2, and pan-cytokeratin. Additional genetic testing was completed to rule out synovial sarcoma for rearrangement involving SS18(SYT; 18q11.2), which was negative.

Treatment was initiated with ifosfamide (3000 mg/m2/m^2^ × 3 days) and doxorubicin (37.5 mg/m^2^ × 2 days) on Children’s Oncology Group protocol ARST0332, ARM D. His clinical course was complicated by mucositis and diarrhea. Because he had an unresectable disease, he initiated adjuvant radiation therapy after the completion of induction chemotherapy. He received proton beam radiation therapy for an initial dose of 45 GyRBE at 1.8 GyRBE per daily fraction, followed by 2 sequential boosts for a total dose of 64.8 GyRBE at 1.8 GyRBE per daily fraction, which started on week 4. He was treated with a 3-field proton beam arrangement (anterior-posterior/posterior-anterior and superior anterior oblique beam). He subsequently completed consolidation chemotherapy per the Children’s Oncology Group protocol ARST0332 several months later for a total of 5 cycles (total doxorubicin dose was 375 mg/m^2^). The patient underwent baseline pulmonary function testing and ECHO after the completion of treatment.

### Follow Up

After 5 years and 10 months, the patient has had no evidence of disease. However, the patient has developed late sequelae because of his chemoradiation for his mediastinal sarcoma. He has developed progressively worsening shortness of breath at rest and with exertion. Additionally, he was diagnosed with asthma and obstructive sleep apnea. Owing to his ongoing symptoms, the patient became established with a cardiologist for heart failure with preserved ejection fraction and chronic pericarditis. The patient underwent subsequent testing for the management of these toxicities. A pulmonary function test revealed a mild restrictive component. An ECHO revealed 55% to 60% left ventricular ejection fraction with mild aortic insufficiency and tricuspid regurgitation. Follow-up staging CT scans were completed. Although it revealed no evidence of recurrent disease or other distant visceral metastases, there was an irregular fibrotic stranding in both the lower lungs and medial segment of the right middle lobe that extended to the medial right and left hemidiaphragm. For pericarditis evaluation, a heart scan with gadolinium indicated small biventricular chamber sizes with mild globally depressed quantitative function in 51% left ventricular ejection fraction and 50% right ventricular ejection fraction. There was mild tricuspid and mitral regurgitation, and the atria were normal.

An exacerbation of the patient’s shortness of breath and lower extremity edema prompted him to be evaluated in the emergency department, for which the patient was placed on diuretic therapy. A few days later, his cardiologist ordered a cardiac MRI, which revealed no myocardial scars but significant hyper-enhancement of the pericardium, suggesting active inflammation and constrictive pericarditis. There was a pericardial area of increased thickening anterior to the right ventricle (6-7 mm). Additionally, bilateral pleural effusions were present. As a result, he was treated with 4 months of high-dose corticosteroids and colchicine for constrictive pericarditis. A repeat cardiac MRI after this medical treatment revealed ongoing pericardial thickness and enhancement and ventricular interdependence. Despite no radiographic evidence of response to medical therapy, the patient had significant improvement in symptoms, including resolution of shortness of breath and edema. His last evaluation showed no evidence of clinical or radiographic disease recurrence. Approximately 6.5 years out from his treatment, he has ongoing care with multiple cardiac specialists, given concern for restrictive and constrictive heart disease and chronic pericarditis and inflammation in the setting of chemoradiation toxicity. As he is classified as having Stage D Class III/IV Constrictive/Restrictive cardiomyopathy related to radiation and chemotherapy, he is being further evaluated for heart transplantation.

## Discussion

Mediastinal soft tissue sarcomas are rare and often associated with a poor prognosis when deemed unresectable. In a retrospective of 74 primary sarcomas of the mediastinum, complete surgical resection was associated with higher rates of survival (5-year overall survival [OS] 49%) in comparison with incomplete resection or no resection (3-year OS 18%) (*P* = .0016) [2]. High-grade sarcomas also worsened overall survival versus low-grade tumors (5-year OS 22% vs 66%, *P* = .05) [2]. In a National Cancer Database analysis of 974 patients, treatment approaches and their associated outcomes were assessed [10]. Surgery and radiation had significantly better OS than treatments not including surgery, such as chemoradiation, radiation alone, chemotherapy alone, or no treatment [10]. The 5-year overall survival was highest at 40.1% in patients receiving surgery followed by radiation therapy compared with 8.5% in patients who received radiation or chemotherapy alone. [10] The Children’s Oncology Group ARST0332 evaluated a risk-based treatment for soft tissue sarcoma in patients younger than 30 [11]. There were 2 arms for patients deemed intermediate risk (unresectable disease of any size or grade or > 5 cm nonmetastatic R0 or R1 high-grade tumors). Patients with > 5 cm nonmetastatic, high-grade tumors who underwent R0 or R1 resection received adjuvant chemoradiation. Patients with unresectable or a high-grade tumor > 5 cm where a delayed resection was planned received neoadjuvant chemoradiation. The 5-year event-free and overall survival of intermediate-risk patients were 65% and 79.2%, respectively [11]. Of intermediate-risk patients, unresectable disease predicted poor 5-year EFS (29.6% R2/no resection vs 70.5% for R0/R1, *P* < .001) [11].

**Figure 1.**
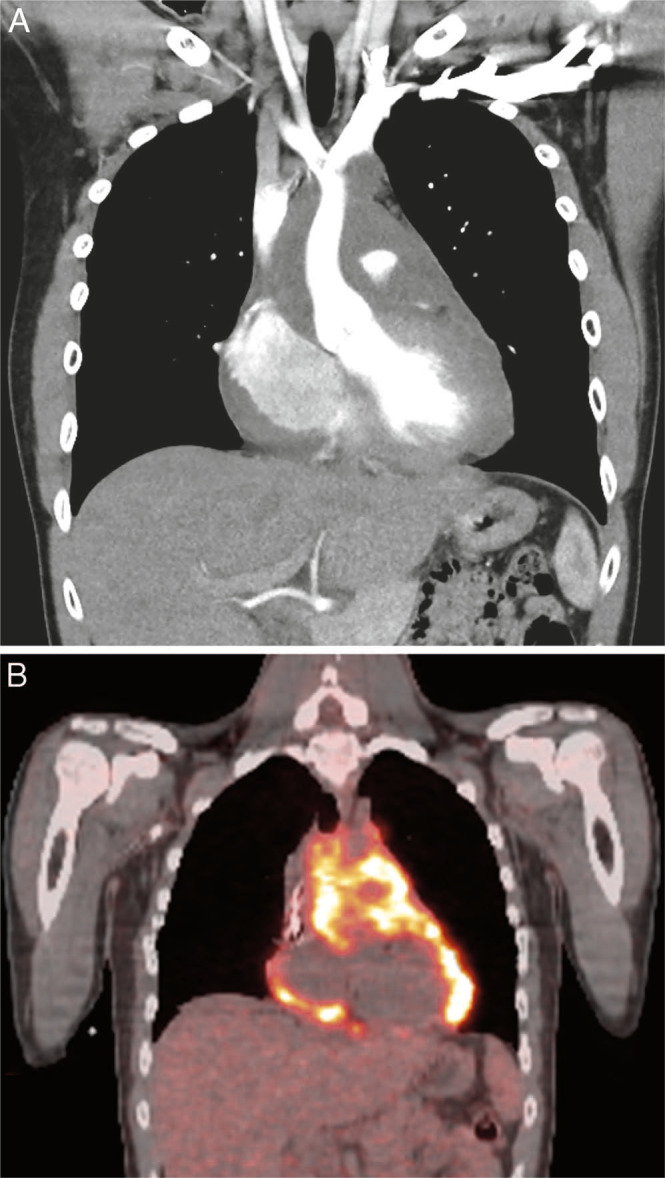
Coronal view of computed tomography scan (A) and positron emission tomography (PET) scan (B) demonstrating extensive sarcoma involving the mediastinum and pericardium.

For this case, to maximize the chances of survival and minimize the dose to surrounding structures, the tumor was targeted using proton beam therapy to definitive doses, which was challenging to achieve using photon-based techniques (**Figure 1**, **Table**). Delivery of proton beam therapy to the mediastinum can present several challenges. Maintaining electronic equilibrium at the interface of lung and soft tissue and managing target motion is common to all external-beam modalities. With pencil-beam scanning protons, beam-target interplay must be carefully considered as part of the motion management treatment, and changes to the patient habitus throughout treatment must also be monitored. Target coverage can be significantly affected by beam-target interplay during free breathing as the amplitude of internal target motion increases, especially for small targets.

**Figure 2.**
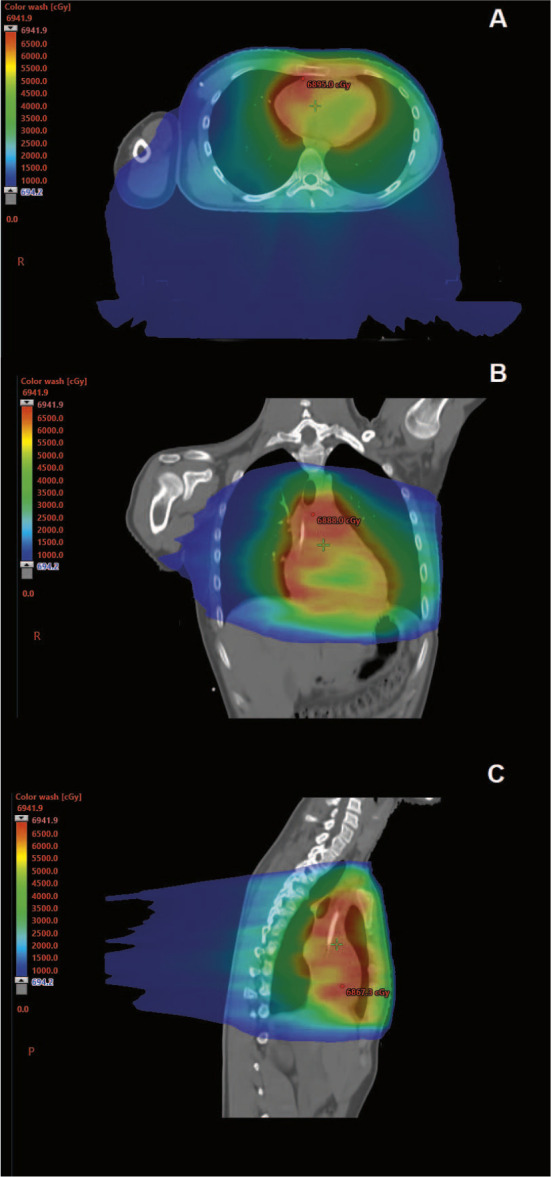

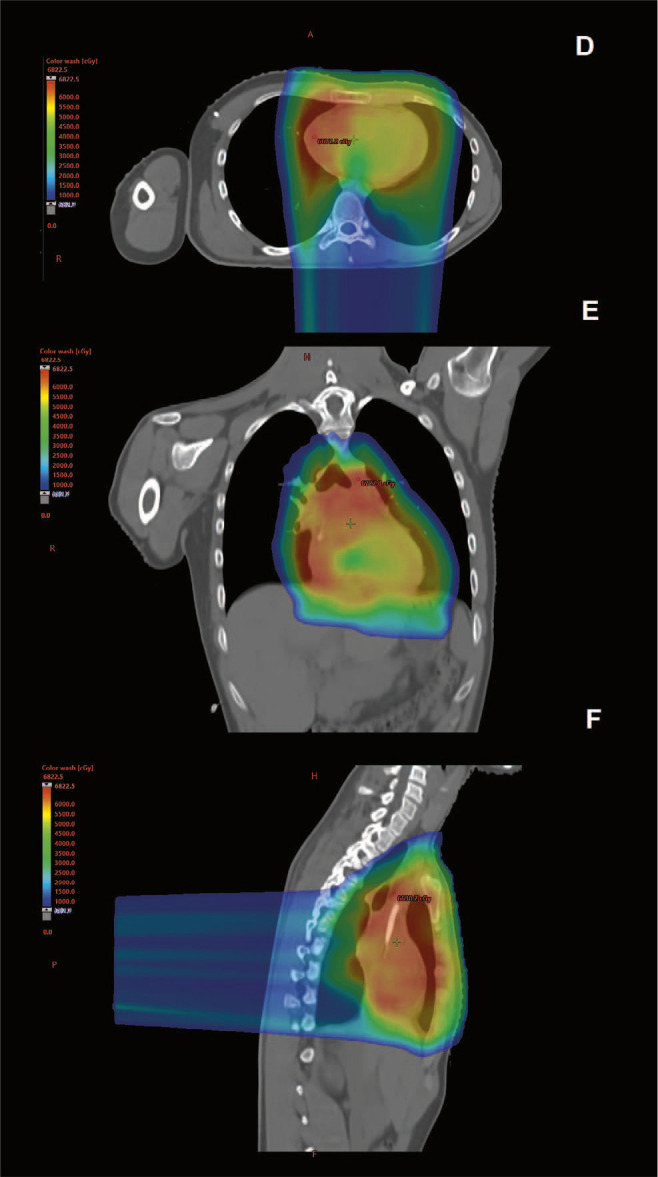
Axial (A and D), coronal (B and E), and sagittal (C and F) of intensity-modulated radiation therapy (A–C) and proton (D–F) dose color-wash for mediastinal soft tissue sarcoma.

**Table.** Dose constraints for organs at risk for IMRT and proton plan.

**Structure**	**Dose constraint**	**IMRT plan value**	**Proton plan value**
CTV High	V100 ≥ 95%	86.0%	30.8%
	D95% ≥ 100%	98.0%	97.3%
	V95% ≥ 99%	99.3%	99.5%
	V115% ≤ 0.5 cm^3^	0	0
	D1% ≤ 110%	106.2%	103.477
	V100% ≥ 99%	86.0%	30.8%
Spinal Cord	V4500 cGy ≤ 0.1%	0%	0%
Total Lung	Mean ≤ 1200 cGy	2864.4 Gy	1753.8 cGy
	V1300 cGy ≤ 30%	89.4%	44.59%
	V2000 cGy ≤ 20%	71.8%	35.97%
Heart	Mean ≤ 2600 cGy	5717.5 cGy	5571.3 cGy
	V2500 cGy ≤ 50%	100%	100%
	V3000 cGy ≤46%	100%	100%
	V4000 cGy ≤ 20%	100%	99.07%
	V5000 cGy ≤ 20%	89.6%	90.56%
Liver	Mean < 2500 cGy	955.2 cGy	514.4 cGy
	V3000 cGy < 60%	8.4%	7.0%
	MVS3000 cGy > 700 cm^3^	1355.7 cGy	1376.7 cGy
Esophagus	Mean < 3400 cGy	3754.4 cGy	2859.7 cGy
	V3500 cGy ≤ 50%	65.0%	34.0%
	V5000 cGy ≤ 45%	24.9%	10.5%
	V5500 cGy ≤ 40%	6.7%	4.7%
	V6000 cGy ≤5%	1.0%	0.2%

**Abbreviation:** IMRT, intensity-modulated radiation therapy.

Consequently, target motions > 1.0 cm require gated proton-beam delivery for most targets at our institution, based on an external marker block resting on the patient’s abdomen. A free-breathing delivery was employed for this case, based on a maximum target respiratory excursion of 0.7 cm determined from a 4-dimensional CT. During the planning work-up before beam optimization, tissue-appropriate overrides to the Hounsfield Units were applied to the lung tissue within 3-5 mm of the radiation target. During optimization, this ensured beamlets at the target boundary would be assigned energy sufficient to cover the target in the case of random patient setup variations and target motions, thereby maintaining a robust electronic equilibrium within the target. During radiation spills, the scanning beam translates rapidly enough that the target would be considered stationary during delivery. However, the scanning beam had a roughly 20% duty cycle delivered in a series of 2s bunches, spaced apart enough that a moving target driven by respiration time scales would be in a slightly different position for each bunch start time. A simulation platform was developed to mimic the impact of this interplay between target and scanning beam motions for various start times and positions based on the patient’s breathing patterns observed during the initial simulation. Additional CT scans were performed during treatment for verification.

Lung effects from radiation, specifically radiation pneumonitis and radiation fibrosis, are significant dose-limiting factors for thoracic radiation for adults and children [12]. The dose to the lungs was a primary consideration for selecting proton beam therapy rather than intensity-modulated radiation therapy. Dosimetric parameters, such as mean lung dose and volume of lung above a threshold (V20 Gy), determined from scrupulous evaluation of adult patients with lung cancer, are routinely used to assess the risk of radiation pneumonitis in children and adults [12]. While parameters are frequently used, they have low predictive value, as approximately 46% to 71% of patients with high-risk parameters do not experience radiation pneumonitis, and 7% to 16% of patients with low or moderate risk experience radiation pneumonitis [12–14]. In contrast to adults, there have been lesser attempts to evaluate radiation pneumonitis in children and young adults. Hua et al [15] evaluated the risk of radiation pneumonitis in 122 patients with sarcoma of Hodgkin lymphoma receiving 3-dimesnional conformal radiation or intensity-modulated radiation therapy to the chest. The 2-year incidence of symptomatic radiation pneumonitis was 9.1% in this cohort. Two variables, lung V24 Gy and bleomycin chemotherapy, correlated with radiation pneumonitis risk. Although the patient in this case report did not receive bleomycin, the patient received a high dose to the mediastinum. The intensity-modulated radiation therapy plan would have the following results: mean lung dose = 29 Gy, V13 Gy = 89%, and V20 Gy = 72%. With proton beam therapy, the mean lung dose was lowered by roughly 40%, and the V13 Gy and V20 Gy were lowered relatively by 50%.

Given the extensive and unresectable nature of his sarcoma involving the mediastinum and pericardium in our patient, high-dose radiation was delivered with proton beam therapy. Given the patient’s poor prognosis at the time of diagnosis and treatment, a full radiation dose of up to 64.8 Gy was delivered to the heart to provide a chance for cure. However, while the patient has no clinical or radiographic evidence of disease 6.5 years from treatment, he is dealing with sequelae of constrictive pericarditis and heart failure with preserved ejection fraction. Morbidity and death due to radiation-induced heart disease, including cardiomyopathy, valvular heart disease, coronary artery disease, and chronic constrictive pericarditis, are well known [16–18]. The pathophysiology of radiation-induced heart failure often occurs in the setting of constriction and restriction with concomitant coronary artery disease and valvular heart disease [16, 19]. The Mayo Clinic performed a retrospective study identifying 12 patients who developed radiation-induced cardiomyopathy requiring cardiac transplantation [16]. However, the primary diagnosis was Hodgkin lymphoma in 8 and non-Hodgkin lymphoma in 4 [16]. The mean interval between RT and cardiac transplantation was 23.2 years (median 25.1 years, range 13.4-31.3 years) [16]. After cardiac transplantation, overall survival at 1, 5, and 10 years was 91.7%, 75%, and 46.7%, respectively [16].

In summary, we demonstrate the successful treatment of unresectable mediastinal high-grade sarcoma with chemotherapy and high-dose proton beam therapy. Despite the therapeutic success of the treatment regimen, the late toxicity of this aggressive approach will need to be addressed.
